# Relationships between non-communicable disease, social isolation and frailty in community dwelling adults in later life: findings from the Hertfordshire Cohort Study

**DOI:** 10.1007/s40520-021-02026-3

**Published:** 2021-11-29

**Authors:** Gregorio Bevilacqua, Karen A. Jameson, Jean Zhang, Ilse Bloom, Nicholas R. Fuggle, Harnish P. Patel, Kate A. Ward, Cyrus Cooper, Elaine M. Dennison

**Affiliations:** 1 MRC Lifecourse Epidemiology Centre, University of Southampton, Southampton General Hospital, Southampton, SO16 6YD UK; 2 NIHR Southampton Biomedical Research Centre, University of Southampton, Southampton, UK; 3 University Hospital Southampton NHS Foundation Trust, Southampton, UK; 4 Medicine for Older People, University Hospital Southampton, Southampton, UK; 5 National Institute for Health Research Musculoskeletal Biomedical Research Unit, University of Oxford, Oxford, OX3 7LE UK; 6 Victoria University of Wellington, Wellington, New Zealand

**Keywords:** Ageing, Multimorbidity, Non-communicable diseases, Older people, Social Isolation, Frailty

## Abstract

**Background:**

Social relationships play a fundamental role in individuals’ lives and health, and social isolation is prevalent among older people. Chronic non-communicable diseases (NCDs) and frailty are also common in older adults.

**Aims:**

To examine the association between number of NCDs and social isolation in a cohort of community-dwelling older adults in the UK, and to consider whether any potential association is mediated by frailty.

**Methods:**

NCDs were self-reported by 176 older community-dwelling UK adults via questionnaire. Social isolation was assessed using the six-item Lubben Social Network Scale. Frailty was assessed by the Fried phenotype of physical frailty.

**Results:**

The median (IQR) age of participants in this study was 83.1 (81.5–85.5) years for men and 83.8 (81.5–85.9) years for women. The proportion of socially isolated individuals was 19% in men and 20% in women. More women (18%) than men (13%) were identified as frail. The number of NCDs was associated with higher odds of being isolated in women (unadjusted odds ratio per additional NCD: 1.65, 95% CI 1.08, 2.52, *p* = 0.021), but not in men, and the association remained robust to adjustment, even when accounting for frailty (OR 1.85, 95% CI 1.06, 3.22, *p* = 0.031).

**Discussion:**

Number of self-reported NCDs was associated with higher odds of social isolation in women but not in men, and the association remained after considering frailty status.

**Conclusions:**

Our observations may be considered by healthcare professionals caring for community-dwelling older adults with multiple NCDs, where enquiring about social isolation as part of a comprehensive assessment may be important.

## Background

Social relationships are important in individuals’ lives and health, and have previously been associated with physical and psychological wellbeing [1]. Social isolation is considered as an objective measure of the scarcity or absence of regular social contacts and relationships with relatives, friends and neighbours and lack of social connection and involvement with the wider society [2–4]. As such, social isolation is distinct from loneliness, which is intended as a subjective, negative evaluation of the discrepancy between one’s desired and actual quantity and quality of social relationships [5–7]. Previous studies reported that social isolation is prevalent and increasing among older adults [8, 9]. This is a growing public health concern, as social isolation has been associated with a number of both physical and psychological adverse health outcomes, such as poor physical capability, myocardial infarction, stroke, depression and mortality [10–16]. Therefore, recent studies have highlighted the importance of developing and implementing interventions aimed at reducing social isolation (as well as loneliness) in older populations [17, 18].

In addition, an increase in life expectancy and a subsequent ageing population have led to a higher prevalence of chronic, non-communicable diseases (NCDs) [19]. The co-existence of two or more NCDs in one patient is defined as multimorbidity [20, 21], a phenomenon that increases with age [22]: a study utilising a survey of members of a health maintenance organisation aged 65 and over, found the average person had 8.7 chronic diseases [23], while a Canadian study reported that the number of chronic diseases varies from 2.8 in young patients to 6.4 among older patients recruited from regional general practices [24]. The World Health Survey carried out between 2002 and 2004 in 70 countries worldwide showed that about 50% of middle-aged (50–64 years) to older (≥ 65 years) adults were multimorbid, having two or more NCDs, approximately a quarter had three, and one tenth have four or more NCDs [25]. A study by Kingston et al. using data from two population-based English cohorts of older adults living in the community (i.e. the English Longitudinal Study of Ageing [ELSA] and the Cognitive Function and Ageing Studies II) reported a 45.7% prevalence of multimorbidity (defined as having two or more NCDs) in 2015 for individuals aged 65–74 years, and estimated that such prevalence might increase to 52.8% by 2035 [26].

While a number of studies focused on the link between multimorbidity and loneliness [27–32], studies looking at potential associations between the number of coexisting NCDs and social isolation are very rare. A recent systematic review of observational studies examining the link between multimorbidity and loneliness, social isolation, and social frailty (i.e. the lack of resources to meet one’s basic social needs) highlighted the lack of studies examining the association between multimorbidity and social isolation [33].

The occurrence of NCDs in older adults is often accompanied by frailty [34, 35], a multi-dimensional geriatric syndrome that can be defined as a state of increased vulnerability resulting from decreased physiological reserves, multi-system dysregulation and limited capacity to maintain homeostasis [36, 37]. Frailty is associated with higher risks of falls, disability, hospitalisation and mortality [38], and it has been reported to predict increased social isolation [39]. It is thus possible that any link between NCDS and social isolation might be mediated by frailty.

In the current study, we, therefore, investigated whether the number of self-reported NCDs is associated with social isolation in a cohort of community-dwelling older adults in the UK. We also sought to explore whether any observed associations were removed by adjustment for the presence of frailty.

## Methods

Participants were recruited from the Hertfordshire Cohort Study (HCS), a population-based sample of men and women born between 1931 and 1939 in Hertfordshire and originally recruited to study the relationship between growth in infancy and the subsequent risk of adult diseases [40, 41]. Between 2019 and 2020, 176 participants from the HCS (94 men and 82 women) were visited at home by a trained fieldworker who administered a questionnaire that included information on medical history, medication use, lifestyle and social isolation. The visits also included measurements of height and weight to calculate body mass index (BMI); grip strength assessed three times for each hand using a Jamar dynamometer (the maximum measurement was used for analysis) [42]; the performance of the Short Physical Performance Battery (SPPB) tests, which included the assessment of gait speed, measured using an eight-foot course with no obstructions for an additional foot at either end. Participants were asked to walk at their customary pace and the time taken was recorded using a stopwatch; the use of assistive devices, such as canes, was permitted if necessary; gait speed was determined by dividing the distance traversed by the time between the first and last step [43].

Social isolation was assessed using the 6-item Lubben Social Network Scale (LSNS-6), which has been validated to assess social networks and social support and to screen for social isolation in older people [44]. The LSNS-6 tool measures the number and frequency of social interactions with friends (three items) and family members (three items). Each answer is assigned a score ranging from 0 (“none”) to five (“nine or more”), and the overall final score ranges from 0 (indicating high isolation or few social resources) to 30 (indicating low isolation or many social resources). Social isolation was defined as a LSNS-6 score < 12, in accordance with Lubben et al. [44]. The LSNS-6 has been shown to have good internal consistency across samples of community-dwelling older adults [44–46].

Number and types of NCDs were assessed by asking the question: ‘Have you been told by a doctor that you have any of the following conditions?’. The following conditions were recorded: high blood pressure, diabetes, lung disease (asthma, COPD, emphysema, chronic bronchitis), rheumatoid arthritis, multiple sclerosis, cancer, vitiligo, depression, Parkinson’s disease, heart disease (heart attack, angina, heart failure), peripheral arterial disease (claudication), osteoporosis, thyroid disease, and stroke. Any other serious illnesses were also recorded.

Frailty was defined as the presence of at least three of the following Fried frailty criteria [38]: unintentional weight loss, weakness, self-reported exhaustion, slow gait speed and low physical activity. Weight loss was assessed asking the question: ‘In the past 3–6 months, have you lost any weight unintentionally? If yes, how much?’. Weakness was defined as a maximum grip strength of < 27 kg for men and < 16 kg for women [47]. Exhaustion was assessed asking the following question: ‘How often in the last week did you feel “everything I did was an effort” or “I could not get going?”’. Participants who responded to feel as described above for either moderate amounts or most of the time were identified as exhausted. Slow gait speed was defined as ≤ 0.8 m/s. Physical activity was assessed by the average amount of time (in minutes per day) spent walking outside, cycling, gardening, playing sports or doing housework in the last 2 weeks. Low physical activity was defined as an activity time in the bottom fifth of the HCS sex-specific distribution (≤ 58 min/day for men and ≤ 90 min/day for women). Frailty assessed using Fried’s criteria has predictive validity for adverse health outcomes, including disability [38, 48].

Smoker status was categorised as never smoked, ex-smoker or current smoker depending on the participants’ answers to the questions ‘Do you currently smoke?’ and ‘Have you ever been a smoker?’. Participants were asked how often they currently drank different types of alcohol (beer, wine, spirits, etc.) and how much they normally drank each time. This was used to estimate their alcohol consumption in units per week. Marital status was also ascertained and dichotomised for analysis as ‘currently married’ and ‘single, divorced, separated or widowed’. Lastly, social class was determined at HCS baseline study (1998) from the participants’ current or most recent occupation for men and never-married women, and of the husband for married women; occupations were classified as non-manual (classes I-IIINM) or manual (classes (IIIM-V) according to the 1990 OPCS Standard Occupational Classification scheme.

### Statistical analysis

Descriptive statistics for continuous variables were expressed as median and interquartile range (IQR); categorical variables were expressed as frequency and percentage. Differences between men and women were assessed using Mann–Whitney tests, Pearson’s *χ*^2^ tests or Fisher’s exact test, as appropriate. Logistic regression analyses were used to examine the associations between the number of NCDs and the social isolation outcome. The regression analyses were undertaken with and without adjusting for the following demographic and lifestyle confounders: age, BMI, social class, marital status, smoker status and alcohol consumption and then further adjusted for frailty. A *p* value of ≤ 0.05 was considered to be statistically significant. The analyses were conducted using Stata version 16.

## Results

Data on NCDs, social isolation, and frailty were available for 176 participants (94 men and 82 women). Table 1 provides the demographic characteristics of the participants. The median (IQR) age of participants in this study was 83.1 (81.5–85.5) years for men and 83.8 (81.5–85.9) years for women. BMI was slightly higher in men (median 27.3, IQR 24.9–29.8) than in women (26.2, 23.7–29.3), although the difference was not statistically significant. The median (IQR) number of NCDs was 2 (1–2) in men and 2 (1–3) in women, and essentially equal proportions of men (19%) and women (20%) were identified as socially isolated on the LSNS-6, while more women (18%) than men (13%) were identified as frail according to Fried’s criteria. None of these differences, however, were statistically significant, the main significant differences being that men were more likely to be currently married compared to women (72% vs 48%, *p* < 0.001), consumed more alcohol units in a week than women (median 2.8, IQR 0.2–8.6 for men and 1.0, 0.0–4.4 for women, *p* = 0.006) and counted fewer subjects who had never smoked (54% of men and 70% of women, *p* = 0.053). Table 1 also presents the number and proportion of participants with each of the NCDs.

**Table 1:** Participants’ characteristics

	Men			Women			*p* value
	*N*	Median	IQR	*N*	Median	IQR	
Age (yrs)	94	83.1	81.5–85.5	82	83.8	81.5–85.9	0.627
Height (cm)	94	171	168–175	81	158	153–162	< 0.001
Weight (kg)	91	79.8	74.5–85.8	82	66.3	56.8–74.8	< 0.001
BMI (kg/m^2^)	91	27.3	24.9–29.8	81	26.2	23.7–29.3	0.206
Alcohol consumption (units per week)	94	2.8	0.2–8.6	82	1	0.0–4.4	0.006
Number of NCDs	94	2	1–2	82	2	1–3	0.846

	Total *N*	*N*	%	Total *N*	*N*	%	*p* value
High blood pressure	94	56	60	82	52	63	0.602
Diabetes	94	18	19	82	18	22	0.646
Lung disease	94	17	18	82	10	12	0.279
Rheumatoid arthritis	94	2	2	82	4	5	0.419
Multiple sclerosis	94	0	0	82	0	0	–
Cancer	94	24	26	82	14	17	0.174
Vitiligo	94	2	2	82	0	0	0.499
Depression	94	3	3	82	8	10	0.073
Parkinson’s disease	94	1	1	82	0	0	1.000
Heart disease	94	36	38	82	15	18	0.004
Peripheral arterial disease	94	0	0	82	0	0	–
Osteoporosis	94	8	9	82	22	27	0.001
Thyroid disease	94	3	3	82	9	11	0.041
Stroke	94	7	7	82	10	12	0.287
Currently married	94	68	72	82	39	48	< 0.001
Social class^a^	88			82			0.470
I–IIINM		37	42		39	48	
IIIM–V		51	58		43	52	
Smoker status	94			81			0.053
Never		51	54		57	70	
Ex		41	44		22	27	
Current		2	2		2	2	
Lubben Social Network Scale < 12	94	18	19	82	16	20	0.951
Fried frailty	84	11	13	78	14	18	0.393

^a^Data obtained from the first pass of the HCS study (1998)

Table 2 displays relationships between the number of NCDs and social isolation. There was no association between the number of conditions and being isolated in men, before or after adjustment. In contrast, a greater number of NCDs was associated with higher odds of being isolated in women in the unadjusted model (OR per additional NCD 1.65, 95% CI 1.08, 2.52, *p* = 0.021). This association persisted after adjustment for confounders, i.e. age, BMI, social class, marital status, smoker status and alcohol consumption (OR 1.93, 95% CI 1.11, 3.34, *p* = 0.020), and it remained robust when Fried frailty was added to the model (OR 1.85, 95% CI 1.06, 3.22, *p* = 0.031). Finally, we also considered whether these relationships were altered after adjustment for the presence of anxiety or depression according to the EuroQoL (moderately or extremely anxious/depressed vs not anxious/depressed); associations were similar after adjustment for this (data not shown).

**Table 2:** Number of NCDs as an explanatory variable for social isolation

	Men				Women			
	*N*	Odds Ratio	95% CI	*p* value	*N*	Odds Ratio	95% CI	*p* value
*Unadjusted*	94	0.90	(0.56, 1.44)	0.660	82	1.65	(1.08, 2.52)	0.021
*Adjusted*^*1*^	84	0.90	(0.52, 1.55)	0.699	78	1.93	(1.11, 3.34)	0.020
*Adjusted* + *frailty*^*2*^	76	1.01	(0.56, 1.81)	0.986	74	1.85	(1.06, 3.22)	0.031

^1^Adjusted for age, BMI, social class, marital status, smoker status and alcohol consumption

^2^Adjusted for age, BMI, social class, marital status, smoker status, alcohol consumption and Fried frailty

## Discussion

We have found a high prevalence of social isolation in our population of older community-dwelling older adults, in line with previous estimates for social isolation among older adults ranging between 15 and 40% [49, 50], and virtually identical to the 19% prevalence of social isolation reported in ELSA participants with a mean (SD) age of 70.3 (16.8) years [51]. These data were collected just prior to the start of the COVID-19 pandemic; the prevalence of social isolation is now likely to be even higher. We also found that a greater number of NCDs in women was associated with a higher odds of being isolated, and this association was not affected by the presence of frailty. In contrast, no associations were found between the number of NCDs and being socially isolated in men.

We were interested to consider whether any possible association between the number of NCDs and social isolation could be explained by the presence of frailty after previous work in ELSA that found that social isolation predicted higher frailty levels, and higher frailty levels predicted greater social isolation [39]. In our study, adjustment for frailty did not remove associations between social isolation and NCDs in women, possibly because there were low numbers of individuals living with frailty in our population sample. Our results hence suggest that even before the onset of frailty, having a greater number of NCDs is associated with social isolation in women—but interestingly not in men.

Despite the paucity of literature on the topic, one previous study by Kristensen et al. found that, in a population of German adults with a mean (SD) age of 63.47 (11.44) years, the onset of multimorbidity was actually associated with increased social networks [27]. This diverges from what we found in our study; such discrepancy can be ascribed to the fact that our population sample is significantly older than the one examined by Kristensen and colleagues. As these authors have highlighted, the onset of physical ill health may have caused an increased need for social contact, especially through support and help [27]. This is to some extent corroborated by another study, conducted in New Zealand with participants aged between 35 and 86 years, which reported that patients with multimorbidity tend to describe social networks mainly consisting of family, support groups, and health care professionals [52]. Being considerably older, our participants are very likely to be much beyond the onset of NCDs and may have already lived with two or more conditions for a long time, by which time their social networks may have decreased in size. It must be noted that Kristensen et al. did not examine possible sex differences [27, 33]. Lastly, Tisminetzky et al. reported that, among American participants with an average age of 61 years, individuals with 4 or more comorbidities were more likely to have a limited social network compared to those with one or less conditions [53]. However, the participants in this study were not only notably younger than ours but also hospitalised individuals rather than community-dwelling adults.

The sexual dimorphism of our findings is striking. We found that the number of NCDs was associated with social isolation in women but not in men. It is possible that the number of NCDs is linked to isolation in women only as women tend to have a greater prevalence and incidence of mobility disability than men [54, 55]: it has been previously reported that social isolation is high among adults with disability [56] and that people with disability have fewer friends, less social support, and are more socially isolated than the general population [56–60]. Women reporting NCDs may be affected by different medical conditions to men, specifically those affecting physical performance to a greater extent [56, 57]; for example, arthritis is more common in women, although we could find no statistically significant difference in prevalence of rheumatoid arthritis between the sexes in our sample, possibly due to the low proportion of men and women with this condition. It is also possible that co-existing depression/anxiety may mediate relationships between NCD and social isolation—again we found no evidence of this in our sample.

In our study, we used a simple count of NCDs rather than a complex measure such as the Charlson grading index of comorbidity [61]. A systematic review of measures of multimorbidity found that simple counts of diseases perform almost as well as complex measures in predicting outcomes such as mortality and health care utilisation [62]. In addition, the mechanisms leading from disease to social isolation can vary substantially, as there can be not only physical but also psychological reasons for social isolation. For instance, vitiligo, a skin disease characterised by a total or partial loss of melanocytes, does not cause decreased mobility (as it can instead be the case for stroke and heart disease which may thus account for social isolation); however, vitiligo, as other chronic skin conditions, is often associated with social stigmatisation and lower social acceptance [63, 64], which can in turn lead to social isolation. Similarly, high blood pressure may not directly be associated with social isolation, but medications prescribed to treat this condition may have a number of side effects (e.g. sedation, fatigue, and insomnia) [65], which can hamper one’s social life and thus induce social isolation. Further work including qualitative analysis (rather than complex measures of morbidity) may be beneficial to the investigation of the relationship between multimorbidity and social isolation in this group.

Our study has a number of limitations. Our study population may not be entirely representative of the wider UK population, since all recruited participants were born in the county of Hertfordshire, were still living in their homes, and were all Caucasian. Nevertheless, it has been previously demonstrated that the HCS is representative of the general population with regard to anthropometric body build and lifestyle factors, such as smoking and alcohol intake, which was in line with data found in the European Investigation into Cancer and Nutrition Cohort (EPIC) [66]. In addition, a ‘healthy’ responder bias is evident within the HCS [40]. Social class was determined at the HCS baseline from the participants’ then current or most recent occupation for men and never-married women, and that of the husband for married women: this is a crude assessment which might not be reflective of participants’ actual occupation and, therefore, social class. An additional limitation of this study is the cross-sectional design of most of its analysis. Lastly, NCDs were self-reported and therefore recall bias cannot be ruled out.

However, our study has also a number of strengths. Firstly, the LNS-6 provides a reliable measurement of social isolation; Rasch analysis showed unidimensionality of the overall scale, high person and item reliability and good fit of individual items with only one exception [67]. Secondly, we assessed frailty using the accepted and objective Fried criteria [68]. We are aware that other methods have been developed in order to assess frailty, but existing literature exploring the relationships between frailty and social isolation using different screening tools is limited [69]. Lastly, the HCS is a population of community-dwelling older adults that have been extensively phenotyped and well characterised with regard to lifestyle and past medical history.

## Conclusions

In a cohort of community-dwelling older adults in the UK, we found that self-reported number of NCDs was associated with social isolation in women only, and that this association was not affected by frailty assessed using Fried’s criteria. Healthcare professionals looking after older adults in a community setting might take our observations into consideration when completing Comprehensive Geriatric Assessments, for individuals affected by NCDs. Future studies may benefit from investigating this association longitudinally and in larger populations, and from exploring whether the association is mediated by impaired physical function and mobility disability. Qualitative studies exploring these relationships in greater detail in women would also be extremely valuable.

## Data Availability

The datasets used and/or analysed during the current study are available from the corresponding author on reasonable request.
